# Epilactose as a Promising Butyrate-Promoter Prebiotic via Microbiota Modulation

**DOI:** 10.3390/life14050643

**Published:** 2024-05-18

**Authors:** Beatriz B. Cardoso, Cláudia Amorim, Ricardo Franco-Duarte, Joana I. Alves, Sónia G. Barbosa, Sara C. Silvério, Lígia R. Rodrigues

**Affiliations:** 1 CEB—Centre of Biological Engineering, Universidade do Minho, Campus de Gualtar, 4710-057 Braga, Portugal; beatrizcardoso.ab@gmail.com (B.B.C.); claudiacv23@gmail.com (C.A.); joana.alves@ceb.uminho.pt (J.I.A.); soniabarbosa@ceb.uminho.pt (S.G.B.); sarasilverio@ceb.umimho.pt (S.C.S.); 2 LABBELS—Associate Laboratory, Guimarães, 4710-057 Braga, Portugal; 3 CBMA—Centre of Molecular and Environmental Biology, Universidade do Minho, Campus de Gualtar, 4710-057 Braga, Portugal; ricardofilipeduarte@bio.uminho.pt

**Keywords:** epilactose, prebiotic, gut microbiota, butyrate, diet, fecal fermentation

## Abstract

Epilactose is a disaccharide composed of galactose and mannose, and it is currently considered an “under development” prebiotic. In this study, we described the prebiotic potential of epilactose by in vitro fermentation using human fecal inocula from individuals following a Mediterranean diet (DM) or a Vegan diet (DV). The prebiotic effect of epilactose was also compared with lactulose and raffinose, and interesting correlations were established between metabolites and microbiota modulation. The production of several metabolites (lactate, short-chain fatty acids, and gases) confirmed the prebiotic properties of epilactose. For both donors, the microbiota analysis showed that epilactose significantly stimulated the butyrate-producing bacteria, suggesting that its prebiotic effect could be independent of the donor diet. Butyrate is one of the current golden metabolites due to its benefits for the gut and systemic health. In the presence of epilactose, the production of butyrate was 70- and 63-fold higher for the DM donor, when compared to lactulose and raffinose, respectively. For the DV donor, an increase of 29- and 89-fold in the butyrate production was obtained when compared to lactulose and raffinose, respectively. In conclusion, this study suggests that epilactose holds potential functional properties for human health, especially towards the modulation of butyrate-producing strains.

## 1. Introduction

Modern globalization has led to the increase in processed food production and, consequently, to changes in lifestyle and dietary patterns, which results in negative outcomes for an individual’s health. Therefore, people are now more aware of the importance of a balanced and healthy diet towards the prevention of so-called “noncommunicable diseases” such as diabetes and cancer. In that sense, consumers are now preferring healthier food options and prebiotics play an important role on this matter, as they can be introduced in diets to provide beneficial effects on the gastrointestinal (GI) tract and the cardiometabolism, as well as on the skeletal and nervous system [1]. Prebiotics are currently defined as “a substrate that is selectively utilized by host micro-organisms conferring a health benefit” by the International Scientific Association for Probiotics and Prebiotics [2] and their market is expected to exceed 9.5 billion USD by 2027 [3].

The main prebiotic mechanism of action is the modulation of the gut microbiota, and, consequently, its effects on several physiological functions. Significant changes can lead to undesirable dysbiosis, which can have a considerable impact on both physical and mental health [4]. The use of prebiotics is a proven efficient strategy to maintain, restore, and promote a healthy microbiota, as they are selectively consumed by the intestinal bacteria [5]. In addition, the short-chain fatty acids (SCFAs) and gases produced by prebiotic fermentation are important for creating and maintaining a healthy gut environment [2].

The functional oligosaccharides, such as lactulose, galacto-oligosaccharides (GOSs), and fructo-oligosaccharides (FOSs), are the most well-established prebiotic compounds, as they are widely studied and used as food ingredients. However, less explored carbohydrates have been emerging as possible competitors due to their potential prebiotic effect, such as, for example, epilactose. Epilactose is an oligosaccharide composed of mannose and galactose that can be produced from lactose using the enzyme cellobiose 2-epimerase [6].

The prebiotic potential of epilactose was first suggested based on its ability to induce the in vitro growth of *Bifidobacterium adolescentis*, *Bifidobacterium breve*, and *Bifidobacterium catenalatum*, under mono-culture conditions [7]. Moreover, the in vivo potential of epilactose to promote the proliferation of *Bifidobacteria* and *Lactobacilli*, while bacteria from *Clostridia* or *Bacteroidetes* classes were inhibited, was reported in Wistar-ST rats [8]. Furthermore, the oral administration of epilactose to mice inhibited the conversion of primary bile acids to secondary bile acids, and increased the calcium absorption, while no elevation of the plasma glucose or lipids levels was detected [8,9]. Recently, in vitro epilactose fermentation using human fecal samples from healthy donors on a “normal diet” was reported, showing beneficial microbiota changes (*Bifidobacterium*, *Megamonas*, *Blautia*, and *Phascolarctobacterium* were increased) and the production of important SCFAs, namely, acetate and butyrate, with a well-known anti-inflammatory effect [10]. Furthermore, a study using an in vitro simulation model of the GI food digestion showed that epilactose can resist upper GI tract conditions, thus reinforcing its potential in being used as a prebiotic [11].

In this work, batch culture studies were performed to assess the prebiotic effect of epilactose using an in vitro static model and human fecal inocula from two healthy donors following different diets (Mediterranean vs. Vegan). This model represents a simple, fast, and practical approach for a preliminary study of compounds with a potential prebiotic effect. Furthermore, it was already used with both well-recognized (lactulose, GOS, FOS, and inulin [12,13,14]) and emergent prebiotics, such as raffinose or xylooligosaccharides [12,15,16]. The experiments included the evaluation of important formed metabolites (SCFAs and gases) and, also, high-throughput sequencing (Illumina MiSeq) to study the modifications promoted on the microbiota. Lactulose and raffinose, whose prebiotic effect was previously reported using this methodology [12], were used for comparison purposes. To the authors’ best knowledge, this is the first study on epilactose prebiotic effects comprising different diets and establishing correlations between the metabolites and the microbiota modulation.

## 2. Materials and Methods

### 2.1. Oligosaccharides Source

Epilactose was enzymatically synthesized using the cellobiose 2-epimerase enzyme from *Caldicellulosiruptor saccharolyticus* produced by *Saccharomyces cerevisiae* [17]. After enzymatic production, a mixture containing 87% of epilactose was obtained by a two-step purification methodology using β-galactosidase and yeast treatment [11]. Lactulose and raffinose (analytical grade) were obtained from Sigma-Aldrich (St. Louis, MO, USA).

### 2.2. Fecal Inoculum

Fecal samples were collected from two healthy young Portuguese adult human volunteers: Donor M (coded DM), 25 years old, who was on a non-specific Mediterranean diet, and Donor V (coded DV), 32 years old, who followed a Vegan diet. Both donors were in the same age range (young adults [18]), were non-smokers, had no metabolic or GI diseases, and were not exposed to antibiotics, or pre- or probiotic supplements for at least 3 months before the fecal donation. Voluntary informed consent was obtained from the two donors prior to this study. The stool samples were collected in sterile plastic containers on the morning of the experiment, and stored under refrigerated conditions during the transport to the laboratory to avoid substantial changes in the metabolic profile and bacterial taxa abundance [19]. Then, the fecal samples were 1:10 (*w*/*v*) diluted in anaerobic phosphate-buffered saline (PBS, 0.01 M, pH 7.0) and stored at 4 °C before inoculation, while all the materials were prepared for the experiment. The anaerobic conditions were maintained using 100% N_2_ in the headspace.

### 2.3. In Vitro Experiments Using Human Gut Microbiota

The experiments were performed using an in vitro static batch culture model, as fully described by Amorim and co-workers [15]. Briefly, the batch cultures were run in anaerobic conditions (100% N_2_) at 37 °C for 48 h, in 70 mL serum bottles. The bottles were filled with 40 mL of nitrogen-sparged growth medium at pH 7.0 (peptone water 2 g/L, yeast extract 2 g/L, NaCl 0.1 g/L, K_2_HPO_4_ 40 mg/L, KH_2_PO_4_ 40 mg/L, MgSO_4_.7H_2_O 0.01 g/L, CaCl_2_.6H_2_O 0.01 g/L, NaHCO_3_ 2 g/L, Tween 80 14.8 mL/L, hemin 5 mg/L, vitamin K1 74.1 μL/L, cysteine HCl 0.5g/L, bile salts 0.5 g/L, Na_2_S.9H_2_O 0.8 mM, and resazurin 1 mg/L). The fecal inoculum was added at 11% (*v*/*v*) to obtain a final fecal concentration of ~1 (*w*/*v*) [19]. The substrate solutions (epilactose, lactulose, and raffinose) were filter-sterilized and added at a final concentration of 10 g/L. The anaerobic conditions were attained by pressurizing the bottle’s headspace with N_2_ up to 170 kPa. Samples of the bottle headspace and the fermentation broth were withdrawn at 0, 24, and 48 h, after moderate manual bottle agitation (successive up and down movements) for mixture homogenization. The gas samples were analyzed to assess the H_2_, CO_2_, and CH_4_ contents. The liquid samples were used to measure the pH, and then centrifuged (4000× *g*, 10 min), and the supernatant was analyzed to verify the production of lactate and SCFAs. At the end of the fermentation (48 h), the biomass was collected, washed, and resuspended in PBS (0.01 M, pH 7.0) and stored at −20 °C for further DNA extraction and sequencing analysis. The fermentations were performed in duplicate, using as negative control a blank with no prebiotic addition.

### 2.4. Analytical Methods

The substrate (epilactose, lactulose, and raffinose) consumption was analyzed by high-performance liquid chromatography (HPLC) according to Cardoso and collaborators [11]. The production of lactate and SCFAs were also analyzed by HPLC as previously described [15]. The H_2_, CO_2_, and CH_4_ contents in the headspace samples were evaluated by gas chromatography as reported by Arantes and co-workers [20].

### 2.5. Microbiota Analysis

Total DNA from the two fecal inocula and the in vitro fermentation media (48 h) was extracted from liquid samples using the FastDNA SPIN kit for soil (MP Biomedicals, Solon, OH, USA), according to the manufacturer’s instructions. The DNA samples were then submitted to high-throughput sequencing (16S rRNA gene, V4 region, primer pair 515F-806R) by Illumina MiSeq technology performed at RTL Genomics (Lubbock, TX, USA), as previously detailed by Salvador and co-workers [21]. The primer pair 515F-806R was selected for microbiota analysis since it generally provides greater depth and taxa coverage in environmental samples, including human feces [10,22]. The obtained reads were denoised and cleaned using RTL Genomics standard pipelines. The analysis was performed in duplicate, and the sequences were submitted as FASTQ files at the European Nucleotide Archive under BioProject accession number PRJEB60534.

### 2.6. Sequence Data Analysis

Raw 16S rRNA sequencing reads were checked for quality control using FASTQC software v. 0.11.9. Clean reads were analyzed for taxonomic profiling using USEARCH global alignment program and the RDP classifier [23,24], and clustered into OTUs using the UPARSE algorithm [25,26]. Briefly, this method finds the top 6 matches in the database for a given sequence and assigns a confidence value to each taxonomic level (kingdom, phylum, class, order, family, genus, and species), by taking the number of taxonomic matches that agree with the top match, and then dividing that number by the total number of total matches. After, for validation, the naïve RDP classifier determines the confidence level of each taxonomic classification.

Taxonomic data were further analyzed using PAST v4.03 [27] for correlation analysis, in particular, considering principal component analysis (PCA), non-metric multi-dimensional scaling (nmMDS), and diversity indices calculation.

Redundancy analysis was used to evaluate the relationship between the samples and SCFAs, using RStudio and the package “vegan”. A correlation heatmap employing a Pearson correlation coefficient was built to inspect significant correlations between microbial classification and SCFAs.

## 3. Results

### 3.1. Production of Lactate and Short-Chain Fatty Acids (SCFAs)

**Figure 1:**
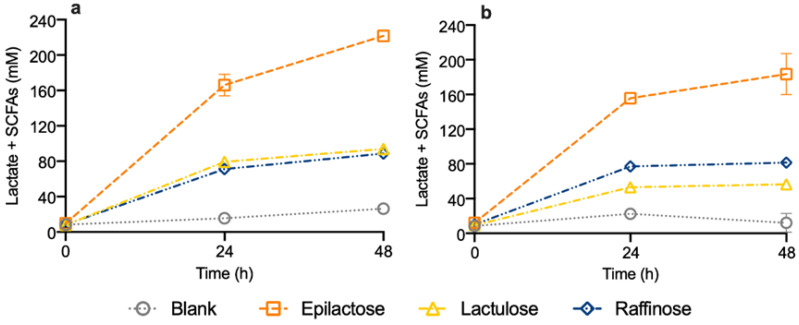
Total production of lactate and SCFAs during 48 h of fermentation using human fecal inocula from donors DM—Mediterranean diet (**a**) and DV—Vegan diet (**b**) in the absence of prebiotics (blank) or in a medium enriched with epilactose, lactulose or raffinose. Results correspond to the mean ± SD (*n* = 2).

Figure 1 presents the production of the total SCFAs, namely, acetate, propionate, and butyrate, and their precursor lactate, during the 48 h of fermentation of the tested prebiotics. It also includes the results obtained for the blank (absence of prebiotic) conditions. In all cases, total consumption of the prebiotics (lactulose, raffinose, and epilactose) was observed after 24 h of fermentation. In general, the profile of lactate and SCFAs during the fermentation was very similar for both donors, and the addition of prebiotics resulted in a significant increase in these metabolites, as expected. Lactulose and raffinose promoted a very similar production of lactate and SCFAs, while epilactose led to their highest accumulation. The maximum concentration of SCFAs and lactate was obtained at 48 h and reached 222 ± 5 mM and 183 ± 24 mM for the DM and DV donors, respectively, when epilactose was added to the medium. In the DM samples, this production was 8.4-fold higher than the control condition (blank) and around 2.4-fold higher than that obtained with the other prebiotics (Figure 1a). For the DV donor, the increase in lactate and SCFAs was 15-, 3.2-, and 2.3-fold higher than the blank, lactulose, and raffinose conditions, respectively (Figure 1b).

**Figure 2:**
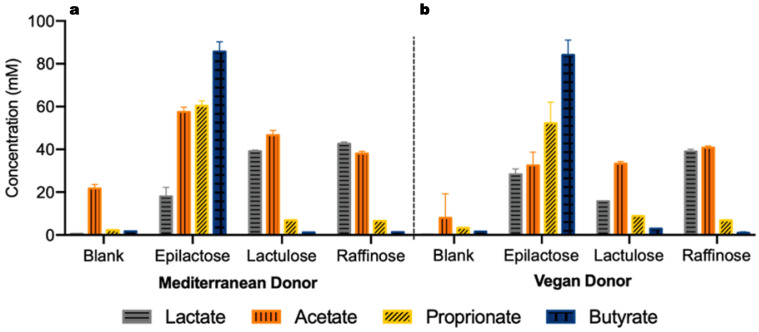
Production of lactate and SCFAs (acetate, propionate, and butyrate) after 48 h of fermentation using human fecal inocula from donors DM—Mediterranean diet (**a**) and DV—Vegan diet (**b**) in the absence of prebiotics (blank) or in a medium enriched with epilactose, lactulose, or raffinose. Results correspond to the mean ± SD (*n* = 2).

The profile of the mixtures containing lactate, acetate, propionate and butyrate produced after 48 h are presented in Figure 2. Again, the differences are more evident between the different prebiotics than between the two donors. As it can be seen, the mixtures obtained with lactulose and raffinose supplementation are rich in lactate and acetate while the addition of epilactose resulted in the highest production of both propionate (DM: 60 ± 2 mM; DV: 49 ± 9 mM) and butyrate (DM: 86 ± 5 mM; DV: 78 ± 6 mM) after 48 h.

### 3.2. pH Variation

**Figure 3:**
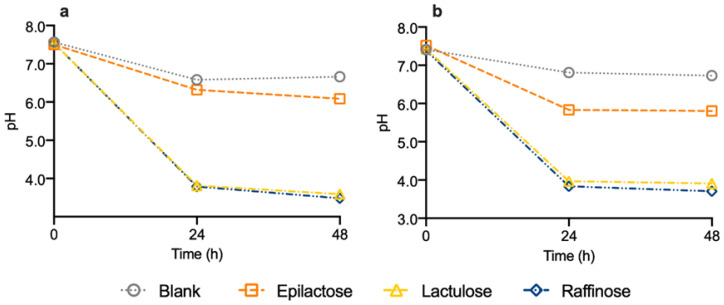
pH variation during 48 h of fermentation using human fecal inocula from donors DM–Mediterranean diet (**a**) and DV—Vegan diet (**b**) in the absence of prebiotics (blank) or in a medium enriched with epilactose, lactulose, or raffinose. Results correspond to the mean ± SD (*n* = 2).

Figure 3 shows the pH variation during the 48 h of fermentation for the three substrates under study and the blank condition. In accordance with the SCFA production, the results showed similar behaviors for both the DM and DV donors. The supplementation with lactulose and raffinose led to a significant reduction in the pH when compared to the control condition. Regarding the epilactose effect, a less pronounced reduction in the pH value was verified. The pH decrease was 1.42 and 1.72 for DM and DV, respectively.

### 3.3. Production of H_2_, CO_2_, and CH_4_

**Figure 4:**
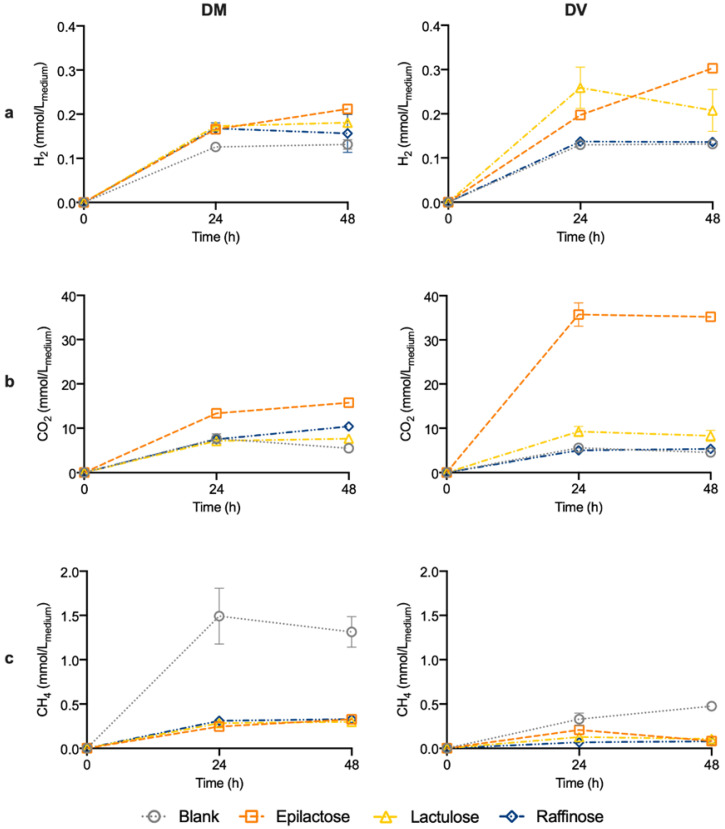
Production of H_2_ (**a**), CO_2_ (**b**), and CH_4_ (**c**) during 48 h of fermentation using human fecal inocula from donors DM (Mediterranean diet) and DV (Vegan diet) in the absence of prebiotics (blank) or in medium enriched with epilactose, lactulose, or raffinose. Results correspond to the mean ± SD (*n* = 2).

Figure 4 illustrates the gas production during the 48 h of fermentation. In general, higher amounts of CO_2_ and H_2_ were produced when prebiotics were added to the medium, for both donors (DM and DV), in comparison with the blank condition. Nevertheless, the most significant differences were found in the supplementation with epilactose, especially for the DV donor. As it can be seen in Figure 4, the fermentation using the fecal inocula from the DV donor resulted in 35.7 ± 2.6 mmol/L of CO_2_ which was 7.8-, 4.2-, and 6.6-fold higher than the blank, lactulose, and raffinose conditions, respectively. For the DM samples, the CO_2_ production in the presence of epilactose does not exceed 2.9-fold higher than the other conditions and reached a maximum of 15.8 ± 0.2 mmol/L. Concerning the CH_4_ production, a significant reduction was observed in comparison with the blank, for the three tested prebiotics. For the DM donor, no differences were found with the addition of the different oligosaccharides and the CH_4_ production was reduced by around 4-fold compared with the blank. The production of CH_4_ in the DV blank sample was significantly low when compared with the DM donor, possibly due to the reduced number of methanogenic bacteria present in the gut microbiota of this donor. Nonetheless, the addition of prebiotics resulted in similar levels of CH_4_. Despite the slightly different profile, the three supplements presented a significant reduction (around five-fold) in CH_4_ when compared with the blank condition at 48 h, for the DV donor.

### 3.4. Intrinsic Differences between Mediterranean and Vegan Inocula

**Figure 5:**
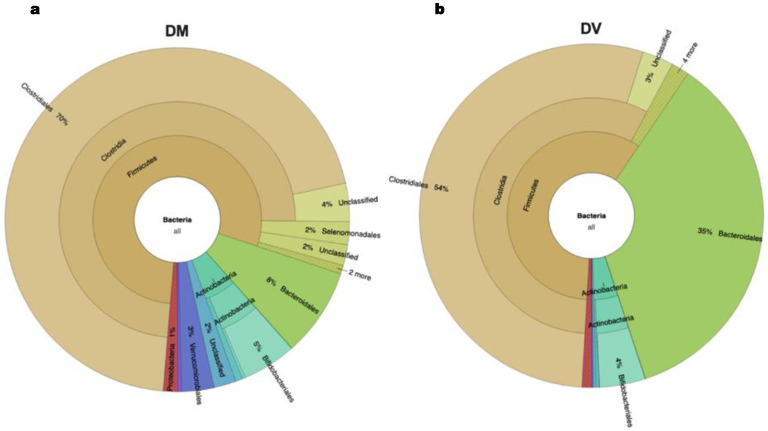
Relative abundance (krona charts) of bacteria determined by 16S rRNA sequencing for DM—Mediterranean diet (**a**) and DV—Vegan diet (**b**) fecal inocula (detailed information in Table S1).

Several studies have already shown that there are differences in the gut microbiota of individuals following an omnivorous or a vegan diet. Figure 5 shows the composition of the fecal inocula obtained from the Mediterranean (DM) and Vegan (DV) donors used in this study. The prevalence of the *Bacteroidetes* phylum, which is considerably higher in the DV donor (35% vs. 8% on DM), is the major difference observed between the two donors following the distinct diets. Another relevant difference was the abundance of *Ruminococcus*, which was 6% in the DM and 3% in the DV donor. The results showed no significant differences between the abundance of *Bifidobacterium* in both donors (5% for DM and 4% for DV, Figure 5). Concerning *Lactobacillus*, the prevalence in the DM sample was 0.3%, while this genus was not detected in the DV sample. In addition, the results also showed a lower presence of pathobionts, such as members of the *Enterobacteriaceae* family (0.3% for DM and 0.09% for DV) and the *Desulfovibrionaceae* family (0.9% for DM and 0.1% for DV) in the donor following the Vegan diet (Table S1). The relative abundance of the *Methanobacteriacea* family was 0.6% for DM and 0.03% for DV (Table S1), which corroborates the difference observed in CH_4_ production.

### 3.5. In Vitro Modulation of the Gut Microbiota

**Figure 6:**
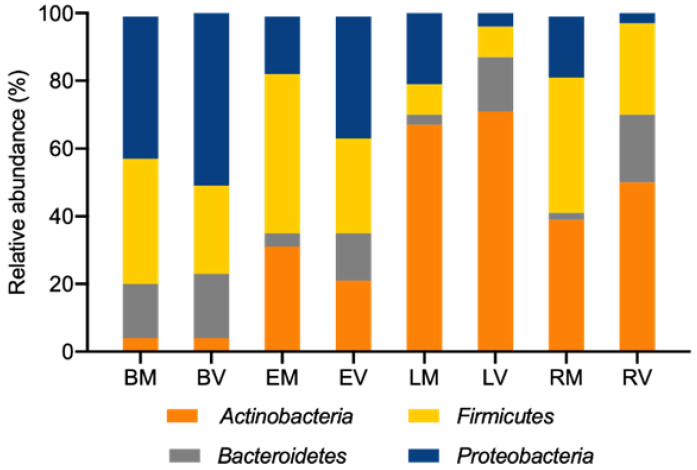
Bacterial composition (relative abundance, %) at the phylum level determined by 16S rRNA sequencing. The *x*-axis shows the blank (B), epilactose (E), lactulose (L), and raffinose (R) conditions for both Mediterranean (M) and Vegan (V) donors.

The microbiota analysis after 48 h of in vitro fecal fermentation showed the typical gut microbiota diversity of healthy humans, as it was mainly composed of four bacterial phyla (Figure 6). The results showed a high similarity between the blank conditions for both DM and DV donors, which demonstrates that, in this case, the diet does not have a significant impact at the phylum level. Figure 6 also highlights the gut microbiota modulation promoted by the supplementation with epilactose, lactulose, and raffinose, which resulted in a pronounced increase in the *Actinobacteria* phylum. Furthermore, the addition of prebiotics also led to a significant reduction in the *Proteobacteria* phylum, where the majority of pathogens associated with intestinal diseases are included.

**Figure 7:**
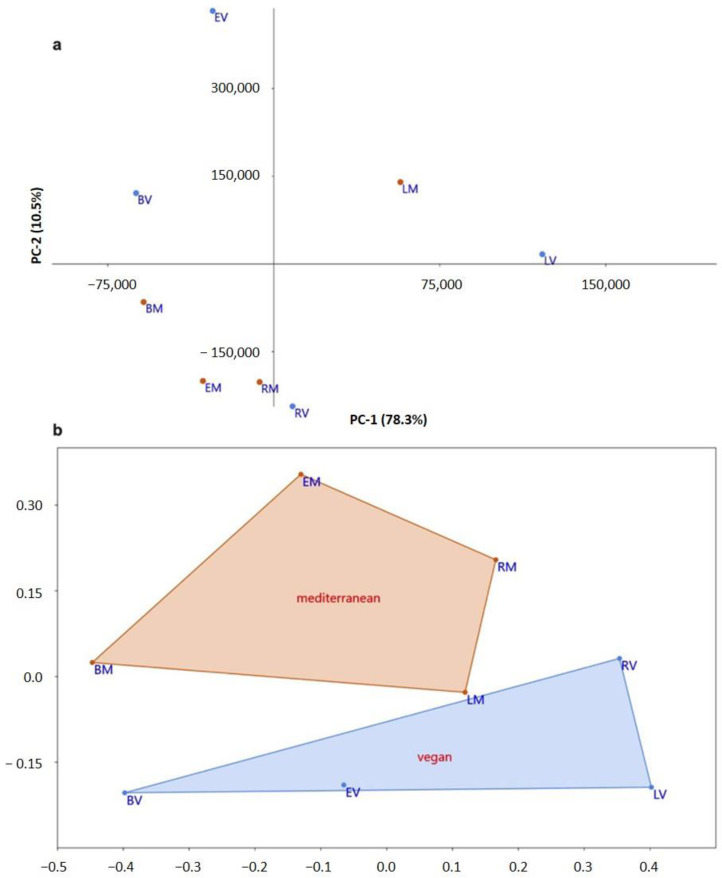
Principal component analysis (**a**) and non-metric multi-dimensional scaling (**b**) of gut microbiota. M: Mediterranean donor, V: Vegan donor, B: blank condition, E: epilactose, L: lactulose and R: raffinose, PC1: first principal component, PC2: second principal component.

A principal component analysis was performed to evaluate the structural changes on the gut microbiota promoted by the use of prebiotics. Figure 7 shows the segregation of the eight analyzed samples, in terms of their differences on the microbiota structure, in the first two components of the PCA. The first and second components (PC-1 and PC-2) explained 78.3% and 10.5% of the observed total variation, respectively, corresponding to 88.8% of the differences observed between the samples.

As Figure 7a illustrates, the supplementation with the three prebiotics increased the structural heterogeneity when compared with the blank condition, for both donors, as shown by the spread of prebiotic samples apart from the blank ones. Additionally, lactulose was the prebiotic that led to the highest difference in the structure of the gut microbiota, as shown by the first component, especially for the Vegan sample, followed by raffinose and epilactose. This last prebiotic showed a large heterogeneity in the Vegan sample, as indicated by the positioning of sample EV in the top of the PCA, as justified by the second PC component.

When comparing the two types of diet (Mediterranean and Vegan), there can be seen almost a clear separation of the different samples in the two PC components, with samples from the Mediterranean diet located almost all in the lower left quadrant of the PCA, with the exception of sample LM, different from the Vegan samples that were located in quadrants I, II, and IV.

Figure 7b shows a non-metric multi-dimensional analysis that was performed to reduce the multidimensional space obtained by the multitude of taxonomic data into a low-dimensional space in order to better compare samples and dieting types. Similarly to the discussed PCA analysis, the two first nmMDS axes sufficiently comprised the variation observed between dieting types, totally separating the Mediterranean and the Vegan samples. In addition, and, again, in accordance with the PCA visualization, it is possible to verify the microbiota structural changes caused by prebiotic samples in comparison with the blank ones, for both diet types.

However, the specific contributors for the verified changes on the microbiota structure cannot be identified only by this type of analysis. In this sense, it is important to discuss other relevant factors. Considering the Shannon_H diversity index, it is possible to see that the LM and LV samples presented lower values and, therefore, a lower bacterial diversity (Table 1), although they were identified as the samples with higher microbiota structural changes by the PCA analysis. This result could possibly be justified by the higher number of reads obtained for these samples, when compared with the blank and the other prebiotics. Furthermore, it was also verified that only two bacterial species were responsible for more than 80% of the reads obtained for the LM and LV samples. In the same line, the Dominance_D index corroborates this observation since higher values indicate the higher dominance of one or few species in a community. On the other hand, the two most abundant bacterial species represented less than 70% and the Dominance_D index was significantly lower for the blank and other prebiotic samples.

**Figure 8:**
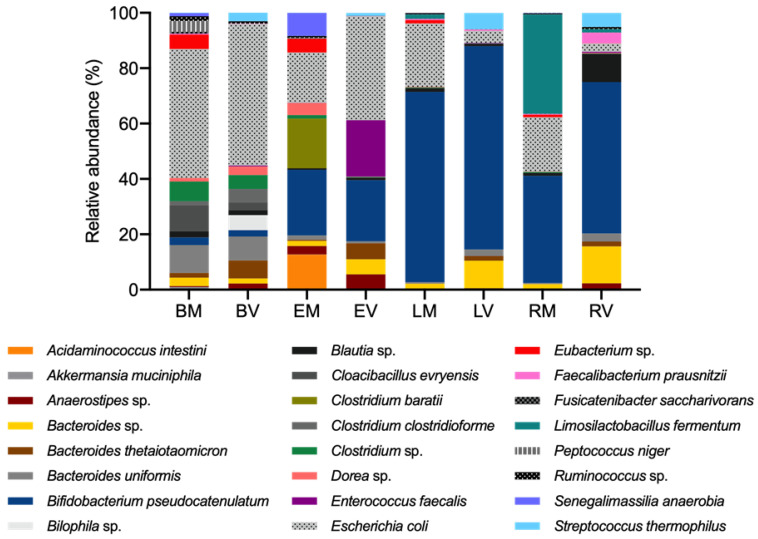
Bacterial composition (relative abundance, %) at the species level determined by 16S rRNA sequencing. The *x*-axis shows the blank condition (B), epilactose (E), lactulose (L), and raffinose (R) for both Mediterranean (M) and Vegan (V) donors. Only micro-organisms assigned to lower taxonomic ranks and with an abundance higher than 3% are represented. Complete taxonomic identification for all taxonomic levels are described in Supplementary Materials.

The biodiversity of a sample is a good parameter with which to evaluate the impact of a prebiotic. Nonetheless, specific changes and proportions of bacterial species are also crucial for assessing the total microbiota modulation promoted by the consumption of prebiotics. Figure 8 shows the distinct modulation of the microbiota promoted by epilactose, lactulose, and raffinose, when compared to the blank condition, for both donors (detailed information in Table S1). As can be seen, differences were found between the DM and DV donors, which might be a result of the different initial composition of the microbiota, as shown in the blank conditions.

One of the major differences observed was the significant increase in *Bifidobacterium pseudocatenulatum*, promoted by the supplementation with the three prebiotics, especially when lactulose was added. The results also showed a higher presence of *Limosilactobacillus fermentum* on the raffinose sample of the DM donor, while it was not detected in the DV sample. As Figure 8 shows, the prebiotic supplementation resulted in a decrease in the abundance of *Escherichia coli*, which suffered a significative reduction with the fermentation of epilactose (51 ± 12% for DM and DV 18 ± 1% for DV), lactulose (36 ± 9% for DM and 90 ± 6% for DV), and raffinose (46 ± 3% for DM and 94 ± 3% for DV). In addition, the fermentation of epilactose also reduced the methanogenic archaea by around 86% for DM and 88% for DV. The supplementation of lactulose and raffinose led to a decrease above 98% for both donors (Table S1).

Considering the most abundant bacterial species promoted by the prebiotic supplementation, a higher diversity was found for epilactose. *Acidaminococcus intestini*, *Clostridium baratii*, *Dorea* sp., and *Senegalimassilia anaerobia* were more stimulated in the Mediterranean sample, while the growth of *Bacteroides* sp., especially *Bacteroides thetaiotaomicron*, and *Enterococcus faecalis,* was higher on the Vegan donor sample. On the other hand, Zhang et al. (2023) highlighted a significant increase in the relative abundance of *Bifidobacterium*, *Megamonas*, *Blautia*, and *Phascolarctobacterium* when using epilactose and human fecal samples from young donors on a non-specified “normal” diet [10].

### 3.6. Metabolite Production vs. Biomass Modulation

**Figure 9:**
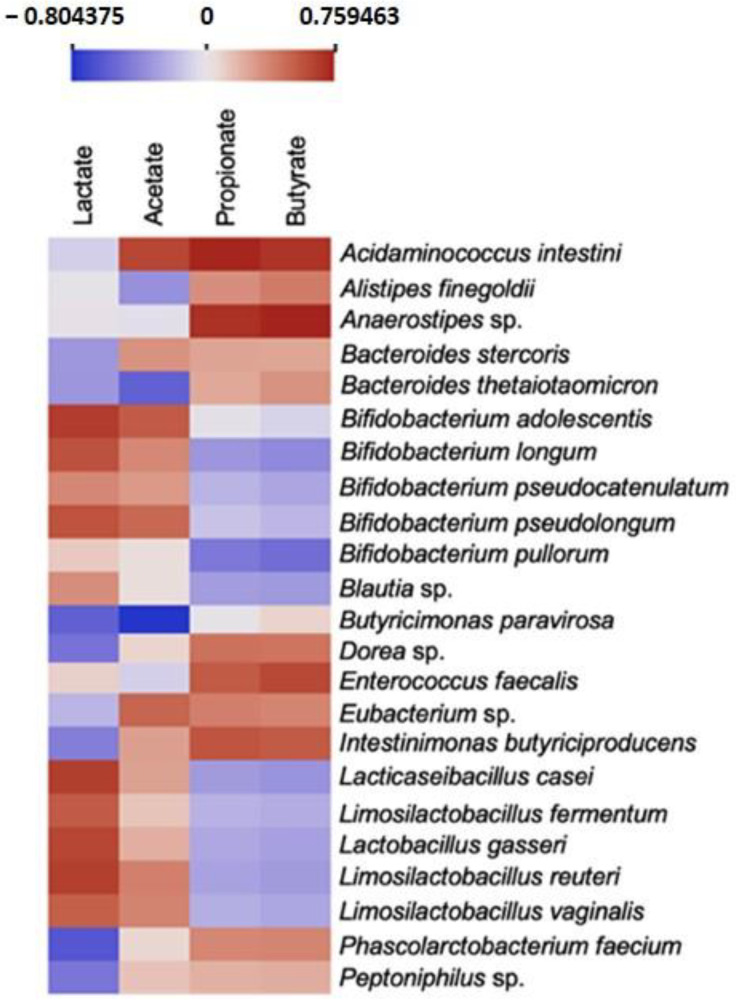
Correlation analysis between bacterial species and lactate, and short-chain fatty acid production. A selection between the most abundant micro-organisms, the most reported as specific metabolite producers, and some species of interest was made. The full heatmap is presented in Figure S1.

A correlation analysis was performed, for the first time, to identify possible associations between the relative abundance of the bacteria species present in the microbiota and the production of lactate and SCFAs (Figure 9). The results showed a strong positive correlation of the *Bifidobacterium* genus and the *Lactobacillaceae* family with the production of both lactate and acetate. On the other hand, strains like, for example, *Acidaminococcus intestini*, *Anaerostipes* sp., *Enterococcus faecalis,* and *Intestinimonas butyriciproducens* were strongly associated with the presence of propionate and butyrate.

## 4. Discussion

It is known that the selective fermentation of prebiotics by the gut microbiota increases the production of SCFAs and lactate, which are crucial beneficial modulators of the metabolic activity and have been linked to several health outcomes [2]. In this study, the fermentation of the three prebiotics significantly promoted the production of lactate and SCFAs, which was around three-fold higher with epilactose. Considering the different donors, similar trends were observed, suggesting that the metabolite production can be more dependent on the type of prebiotic added, rather than on the differences between the gut microbiota of the donors, i.e., the dietary habits.

The total quantification of SCFAs and lactate is generally considered a robust indication of the prebiotic potential. Nonetheless, the proportions in which each SCFA is present in the mixture are also crucial for assessing the potential benefits on the host’s health. Lactulose and raffinose supplementation resulted in mixtures richer in lactate and acetate and these results agree with a previous study performed with the same compounds [12]. On the other hand, the fermentation of epilactose led to the highest production of propionate and butyrate, as already reported by Zhang et al. (2023) [10]. Among the different SCFAs, butyrate stands out due to its crucial role in the maintenance of overall health status. As a gut metabolite, most of the actions of butyrate have an impact on the intestinal environment, namely, as source of energy for colonocytes, in the modulation of the microbiota, and in the enhancement of intestinal mucosa immunity due to its anti-inflammatory properties. These functions may also be further exploited given the potential protective role of butyrate against colon cancer and inflammatory bowel disease [28]. Furthermore, the capacity of butyrate to enter the portal vein and reach other organs revealed a broad range of physiological activities, such as functioning as a metabolic pathways regulator, and having antioxidant, anti-angiogenesis, and anti-obesity properties, and properties against related diseases [29]. These results suggest that both lactulose and raffinose may stimulate the proliferation of lactate- and acetate-producing bacteria, while epilactose probably promotes the microbiota modulation towards propionate- and butyrate-producing strains. These findings demonstrate the diversity of the beneficial effects promoted by the use of different prebiotics, whose consumption can be redirected toward the desired outcome. Additionally, the supplementation with more than one prebiotic can potentially improve the positive effects on human health.

Unlike the in vivo models, where the produced metabolites are rapidly absorbed, in the in vitro models, the produced SCFAs will accumulate and promote changes in the medium pH [30]. These changes are associated with the main health-promoting effects of prebiotics, as they promote the increase in beneficial bacteria populations and a reduction in the intestinal pathogenic agents. In this study, despite the fermentation medium being designed to mimic the distal colon environment and the pH being initially adjusted to 7.0, the medium pH was not further controlled throughout the fermentation to evaluate the global pH changes after 48 h [15]. As expected, when lactulose or raffinose were added, the pH was significantly reduced when compared to the control condition. These results agree with a previous study in which lactulose and raffinose promoted a pH reduction from 7.0 to around 3.5, for two different donors [12]. In the case of epilactose, a slight decrease in pH value was observed. Analyzing the SCFAs results, a greater pH reduction would be expected since epilactose was the compound that led to the higher production of SCFAs. However, the potential presence of unmetabolized culture medium constituents in the epilactose mixture [11] could cause some buffering effects and this probably affected the value of the final pH. Nonetheless, similar results were observed in a recent study reporting the in vitro fermentation of epilactose using fecal samples from six different donors [10].

Apart from the SCFAs, the gut microbiota also produces gases as part of its metabolism, namely, CO_2_, H_2_, and CH_4_ [31]. The evaluation of the prebiotic impact on these compounds’ generation is also important in experiments using fecal inocula. The H_2_ and CO_2_ production is associated with the proliferation and consequent modulation of microbiota promoted by the prebiotic fermentation and, therefore, represents a positive outcome of the use of these compounds. The fermentation of lactulose, raffinose, and, especially, epilactose by the gut microbiota resulted in an increase in H_2_ and CO_2_ production. However, excessive gas production may cause flatulence and bloating problems, which can disincentivize people to consume prebiotics. In this sense, in the case of epilactose, which is an “under development” prebiotic, it is of the utmost importance to perform in vivo human studies to determine the recommended daily dose that minimizes the undesired secondary effects, while maximizing its prebiotic effect. Based on the results herein obtained for gas production, it is suggested that this value should be lower than that already established for lactulose (10 g/day, [32]). High amounts of CH_4_ have been associated with diseases like obesity and colorectal cancer [33], and, therefore, it is also important to study the production of this gas resulting from gut modulation. The addition of the three prebiotics resulted in a significative decrease in CH_4_, which reveals an important outcome of the use of prebiotics, namely, epilactose, as effective promoters of CH_4_ reduction.

The diversity of the gut microbiota is influenced by several factors such as age, geography, diet, pro- or prebiotics consumption, and even metabolic diseases [34]. Dietary habits may contribute to alterations in the gut microbiota composition due to differences in bacteria directly consumed through food but also due to the metabolites that affect the gut ecosystem [35]. The omnivore diet is dependent on the geographical region and differs around the world mainly due to culture and food product availability. A Mediterranean diet is usually rich in legumes, vegetables, meat, fish, and extra virgin olive oil. On the other hand, a Vegan diet is mainly focused on vegetables, legumes, grains, nuts, and seeds, and excludes all foods of animal origin (eggs, meat, fish, and dairy). Additionally, the Vegan diet is also richer in fibers and lower in saturated fat and proteins when compared to the omnivorous diet [36]. These differences among the two diets can result in distinct and characteristic microbiota compositions. In this study, a significantly higher presence of the *Bacteroidetes* phylum in the DV donor was observed. In fact, this is one of the most well-documented impacts of a Vegan diet on the gut microbiota [36]. On the other hand, the DM donor presented a higher abundance of the *Ruminococcus* genus, which was already positively associated with the omnivorous diets [37,38]. Another interesting difference between the two diets was the lower presence of the *Enterobacteriaceae* and *Desulfovibrionaceae* families in the Vegan sample, which is aligned with other studies comparing both diets [39,40,41] that reported the lower presence of pathobionts in individuals following a Vegan diet. The presence of methanogenic archaea was considerably higher in the DM sample which corroborates the difference observed in CH_4_ production. In fact, only about 33% of the world’s population presents methanogens in their gut microbiota [42], which may not be directly related to an individual’s dietary habits. According to the literature, the microbiota of individuals following a Vegan diet may have an increased content of *Bifidobacterium* and *Lactobacillus* genera due to a higher presence of polyphenolic compounds [43]. In this study, no differences were found on the abundance of *Bifidobacterium* in both donors. In fact, comparable results were also reported by other authors [41,44]. In addition, the *Lactobacillus* genus was only detected in the DM sample. A lower content of lactic acid bacteria, including *Lactobacillus* and *Lactococcus*, was also detected by Reiss and collaborators [45] on the fecal microbiota of Vegan donors, when compared to an omnivorous diet. Moreover, it is important to highlight that these kinds of studies are often performed with a low number of donors, which may not fully comprise all the individual differences promoted by the different diets. In addition, the variability in the study design and methods for the microbiota analysis can also compromise a direct comparison between the several reported studies.

For a compound to be considered as a prebiotic, it must promote a health benefit to the host, which can rely on the metabolites produced and environmental changes caused by the utilization of the compound by the microbial groups. Furthermore, if the benefit is measurable and significantly different from a control, it is called a “prebiotic effect” [2]. One example is the modulation of the gut microbiota, which can be assessed by sequencing the 16S rRNA region, thus allowing the study of the bacterial diversity of a certain sample. The supplementation with the three prebiotics resulted in some expected changes when compared with the literature, such as, for example, the significant increase in *Bifidobacterium pseudocatenulatum*, especially when lactulose was added. In fact, other in vitro studies of the prebiotic effect of lactulose have already shown that this compound usually stimulates the growth of the *Bifidobacterium* genus [15]. The higher presence of the *Lactobacillaceae* family on the raffinose sample of the DM donor, represented in this case by the *Limosilactobacillus fermentum*, was also expected. This prebiotic was already reported as a promoter of the *Lactobacillus* growth [12]. The absence of this bacterium in the DV sample is in accordance with the results obtained for the fecal inocula sample of the Vegan donor, where the *Lactobacillaceae* family was not detected. The supplementation with prebiotics resulted also in the decrease in the abundance of the *Proteobacteria* phylum, commonly associated with intestinal pathogens, and the methanogenic population.

The major metabolites of anaerobic fermentation of the GI microbiota are the SCFAs, which play a crucial impact on the host physiology, namely, as sources of energy, signaling molecules, and regulators of gene expression [46]. A recent study showed the production of these metabolites in the presence of epilactose but without establishing a correlation with the changes in the microbiota [10], which is a fundamental aspect to fully understand the prebiotic effects of epilactose. In this sense, a correlation analysis between the relative abundance of the bacteria species and the production of lactate and SCFAs was performed. The results showed a positive correlation between the presence of the *Bifidobacterium pseudocatenulatum* and the production of lactate and acetate. This bacterium was already reported as a producer of these compounds [47] and it was found as one of the most abundant micro-organisms after 48 h of fermentation, especially in the lactulose and raffinose samples, being probably the greatest contributor for acetate and lactate production in these samples. Furthermore, other less abundant species from the *Bifidobacterium* genus and *Lactobacillaceae* family were also positively correlated with the production of both lactate and acetate, as expected since they are well-known as producers of these compounds [48]. Considering all the samples, the production of butyrate was mainly associated with *Acidaminococcus intestini*, *Anaerostipes* sp., *Enterococcus faecalis,* and *Eubacterium* sp., which is well-aligned with the literature [49,50,51,52]. The bacterium *Bacteroides thetaiotaomicron* was recently reported as a propionate producer but also a promoter of the growth of butyrate-producing strains [53] and our results showed a positive correlation between this bacterium and the production of propionate and butyrate. The supplementation with epilactose resulted in the higher production of butyrate, for both donors. In this sense, when analyzing the correlation results and the most abundant species, it is possible to identify the potential one responsible for the butyrate production. In the Mediterranean donor (EM sample), the bacterium *Acidaminococcus intestine*, already highlighted as a reported butyrate producer, was highly promoted. Additionally, *Bacteroides stercoris*, *Intestinimonas butyriciproducens,* and *Peptoniphilus* sp., also reported as butyrate producers [54,55,56], were exclusively present in this sample. Regarding the Vegan donor (EV sample), we hypothesize that *Enterococcus faecalis*, one of the most abundant, and the two exclusive bacteria *Alistipes finegoldii* [57] and *Butyricimonas paravirosa* [58], were the greater contributors for the production of butyrate in this sample.

Considering the results herein gathered, epilactose presents the highest prebiotic potential (when compared to lactulose and raffinose) to stimulate the butyrate-producing strains and boost the butyrate production. In this line, it is important to highlight the potential of epilactose to be used as a prebiotic to help restore the microbiome balance in patients with Crohn’s disease, since it was found that the loss of butyrate-producing bacteria contributes to the proliferation of this disease [59].

## 5. Conclusions

Lactate, SCFAs, and CO_2_ production, together with the microbiota analysis, confirmed the high prebiotic potential of epilactose. The use of fecal donors following a different diet suggested that the prebiotic effect of epilactose may be independent of dietary habits, thus enlarging its potential in promoting health benefits in a vast community. When compared with lactulose and raffinose, the in vitro fermentation of epilactose resulted in the highest production of butyrate, which is an important metabolite due to its benefits for the gut and systemic health. This result was corroborated by the microbiota modulation, especially the increase in the abundance of butyrate-producing strains. This study strongly suggests that epilactose holds potential functional properties for human health. Despite these important pioneer results, in vitro and in vivo studies with more donors and different diets should be conducted in order to obtain robust conclusions about epilactose as a prebiotic with a beneficial action on gut applications.

## Figures and Tables

**Table 1:** Parameters used to evaluate the bacterial diversity and heterogeneity of the gut microbiota. M: Mediterranean donor, V: Vegan donor, B: blank condition, E: epilactose, L: lactulose, and R: raffinose.

Sample	Total of Reads	Dominance_D	Shannon_H	Most Abundant Bacterial Species (Reads)
BM	100,403	0.1631	2.768	*Escherichia coli* (37,670) *Bacteroides uniformis* (8179)
BV	131,914	0.2162	2.438	*Escherichia coli* (58,456) *Bacteroides uniformis* (9935)
EM	125,255	0.1261	2.478	*Bifidobacterium pseudocatenulatum* (27,110) *Escherichia coli* (20,683)
EV	194,509	0.2144	1.969	*Escherichia coli* (68,361) *Bifidobacterium pseudocatenulatum* (40,144)
LM	187,335	0.4654	1.303	*Bifidobacterium pseudocatenulatum* (121,086) *Escherichia coli* (40,289)
LV	251,646	0.5189	1.245	*Bifidobacterium pseudocatenulatum* (178,591) *Bacteroides* sp. (24,144)
RM	148,596	0.2873	1.73	*Bifidobacterium pseudocatenulatum* (53,678) *Limosilactobacillus fermentum* (49,790)
RV	123,094	0.304	1.931	*Bifidobacterium pseudocatenulatum* (62,662) *Bacteroides* sp. (15,257)

## Data Availability

The authors confirm that the datasets supporting the findings and conclusions of this study are available within the article and its supplementary information file. Additional data can be provided upon request.

## Supplementary Materials

The following supporting information can be downloaded at: https://www.mdpi.com/article/10.3390/life14050643/s1, Figure S1: Full heatmap of the correlation analysis between bacterial species and lactate and short-chain fatty acid production; Table S1: Microbial composition until the family level of gut microbiota cultures from DM (Mediterranean diet donor) and DV (Vegan diet donor), supplemented with epilactose, lactulose, or raffinose. Variation in color intensity reflects the relative abundance of microbial groups, from light color, less abundant, to dark color, more abundant. Duplicates are represented by (*a*) and (*b*).
