# Supplementary material for: Epilactose as a Promising Butyrate-Promoter Prebiotic via Microbiota Modulation
Source: Life (Basel). 2024 May 18;14(5):643. doi: 10.3390/life14050643 (PMC11123345; doi:10.3390/life14050643)
Supplement: Supplementary file 1 [file life-14-00643-s001.zip › Cardoso et al_Supplementary Material.pdf]

Epilactose as a promising butyrate-promoter prebiotic via microbiota modulation

Beatriz B. Cardoso,[a] Cláudia Amorim,[a,b] Ricardo Franco-Duarte,[c] Joana I. Alves,[a,b] Sónia G. Barbosa,[a,b] Sara C. Silvério,[a,b*] Lígia R. Rodrigues[a,b]

[a] CEB - Centre of Biological Engineering, Universidade do Minho, Campus de Gualtar, 4710-057 Braga, Portugal

[b] LABBELS - Associate Laboratory, Guimarães, Braga, Portugal

[c] CBMA - Centre of Molecular and Environmental Biology, Universidade do Minho, Campus de Gualtar, 4710-057 Braga, Portugal

*Corresponding author: Sara C. Silvério. E-mail: sarasilverio@deb.uminho.pt

Supplementary Material

Table S1 Microbial composition until the family level of gut microbiota cultures from DM (Mediterranean diet donor) and DV (Vegan diet donor), supplemented with Epilactose, Lactulose or Raffinose. Variation in color intensity reflects the relative abundance of microbial groups, from light color, less abundant, to dark color, more abundant. Duplicates are represented by (*a*) and (*b*)

Fig. S1 Full heatmap of the correlation analysis between bacterial species and lactate and short-chain fatty acids production
