# Supplementary figures and images for: Epilactose as a Promising Butyrate-Promoter Prebiotic via Microbiota Modulation
Source: Life (Basel). 2024 May 18;14(5):643. doi: 10.3390/life14050643 (PMC11123345; doi:10.3390/life14050643)

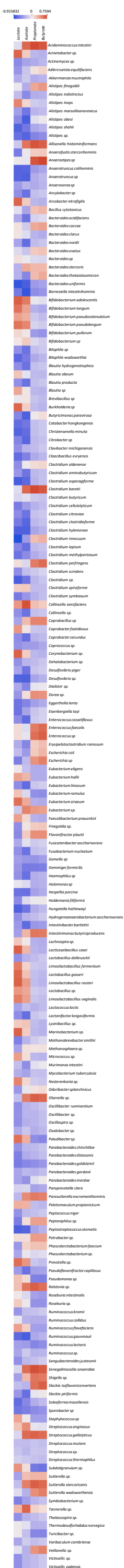

Supplement: Supplementary file 1 [file life-14-00643-s001.zip › Supplementary Material_Fig. S1.pdf]
